# Sequential composite BIMA grafting for 3v-CAD: factors that predict successful outcome of the one-inflow and two-inflow revascularization techniques

**DOI:** 10.1007/s11748-024-02022-0

**Published:** 2024-03-20

**Authors:** Terézia B. Andrási, Alannah C. Glück, Ildar Talipov, Lachezar Volevski, Ion Vasiloi

**Affiliations:** 1 Department of Cardiac and Cardiovascular Surgery, Philipps University of Marburg, Baldingerstrasse 1, 35041 Marburg, Germany; 2 Department of Cardiac Surgery, Cardiac Center, Rotenburg an Der Fulda, Germany; 3 School of Medicine, Philipps University of Marburg, Marburg, Germany; 4 Department of Cardiac Surgery, University of Basel, Basel, Switzerland

**Keywords:** Triple-vessel coronary artery disease, Surgical revascularization, Operative technique, BIMA T-Graft, Two-inflow, Functional mitral valve regurgitation, MACCE

## Abstract

**Objective:**

The effect of one-inflow and two-inflow coronary surgical revascularization techniques inclosing skeletonized double mammary artery (BIMA) as T-graft on outcome is studied.

**Methods:**

Early ad mid-term outcome of complete BIMA revascularization (C-T-BIMA) versus left-sided BIMA with right-sided aorto-coronary bypass (L-T-BIMA + R-CABG) is quantified and analyzed by multivariate logistic regression, Cox-regression, and Kaplan–Meier analysis in a series of 204 consecutive patients treated for triple-vessel coronary disease (3v-CAD).

**Results:**

The L-T-BIMA + R-CABG technique (*n* = 104) enables higher number of total (4.02 ± 0.87 vs. 3.71 ± 0.69, *p* = 0.015) and right-sided (1.21 ± 0.43 vs. 1.02 ± 0.32, *p* = 0.001) coronary anastomoses, improves total bypass flow (125.88 ± 92.41 vs. 82.50 ± 49.26 ml, *p* < 0.0001) and bypass flow/anastomosis (31.83 ± 23.9 vs.22.77 ± 14.23, *p* = 0.001), and enhances completeness of revascularization (84% vs.69%, *p* = 0.014) compared to C-T-BIMA strategy (*n* = 100), respectively.

Although the incidence of MACCE was comparable in the two groups (8% vs.1.2%, *p* = 0.055), the progression of functional mitral regurgitation (FMR) was significantly lower after L-T-BIMA + R-CABG, then after C-T-BIMA (47% vs.64%, *p* = 0.017).

The use of C-T-BIMA-technique (HR = 4.2, *p* = 0.01) and preoperative RCA occlusion (HR = 3.006, *p* = 0.023) predicted FMR progression, whereas L-T-Graft + R-CABG technique protected against it (X^2^ = 14.04, *p* < 0.0001) independent of the anatomic and clinical complexity (Syntax score I: HR = 16.2, *p* = 0.156, Syntax score II: HR = 1.901, *p* = 0.751), of early- (0.96% vs.2%, *p* = 0.617) and mid-term mortality (5.8% vs.4%, *p* = 0.748) when compared to C-T-BIMA, respectively.

**Conclusions:**

The two-inflow coronary revascularization by L-T-BIMA + R-CABG better protects against FMR progression without increasing MACCE and mortality. Older patients with RCA occlusion and reduced LV-EF benefit most from the two-inflow L-T-BIMA + R-CABG technique. Younger 3v-CAD patients with normal LV-EF can preferentially be managed with the one-inflow C-T-BIMA; however, long-term outcome remains to be revealed.

## Introduction

Triple-vessel coronary artery disease (3v-CAD) is more successfully treated by surgical revascularization than by catheter intervention [1] and revascularization to all three major vascular regions is an independent predictor of improved short- and long-term survival after CABG [2–4]. In situ left internal mammary artery (LIMA) grafting to the left anterior descending coronary artery (LAD) [5] and obtaining two conduits from a single sternotomy [6, 7] result in an additional survival benefit of bilateral IMA (BIMA) [8], especially by grafting the right IMA (RIMA) as T-graft to the left coronary system [9–11].

However, RIMA’s length might be insufficient to target all inferior coronary vessels [12, 13]. A single IMA inflow might also be insufficient to achieve maximal myocardial perfusion [14] and using only a T-graft has the potential of losing bypass flow because of the competitive flow of the native circulation [11, 13].

Ischemic cardiomyopathy and myocardial infarct size [15] can subsequently affect the geometry of the mitral valve apparatus, causing functional mitral regurgitation (FMR). Both left ventricular dysfunction and mitral annular dilatation were shown to be associated with FMR progression after CABG [16]. Even a mild degree of FMR portends substantial risk of cardiovascular mortality after acute myocardial infarction [17].

**Fig. 1:**
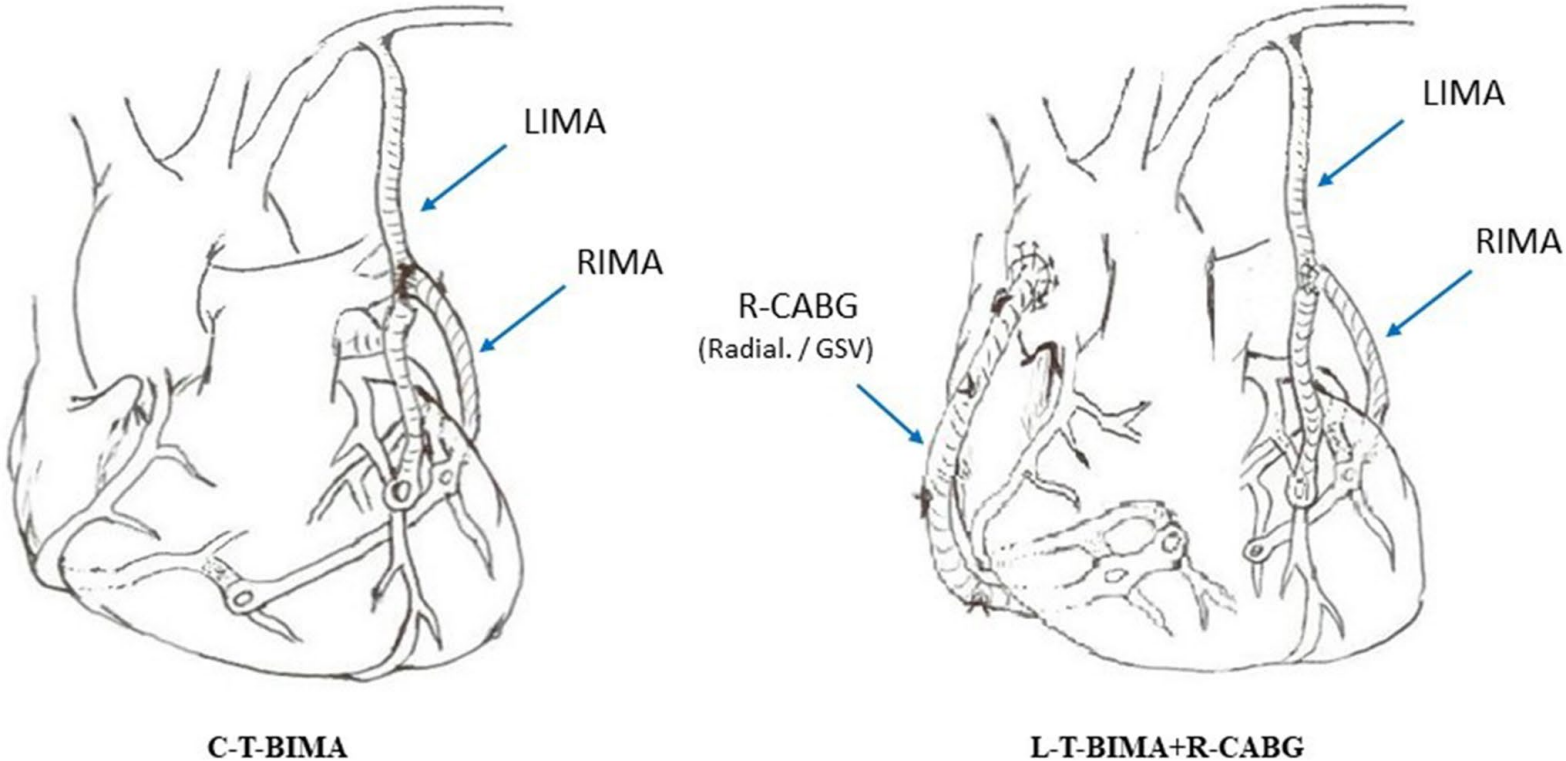
Operative procedures. Schematic representation of the one-inflow in situ fully arterial sequential composite C-T-BIMA technique and of the two-inflow left-sided arterial sequential composite (L-T-BIMA) with separate right-sided CABG (R-CABG) technique

The present study aims to determine the influence of two different coronary surgical revascularization techniques featuring skeletonized BIMA as T-graft (Fig. 1) on the postoperative outcome including atrioventricular valve function.

## Methods

This is a retrospective study on 3v-CAD patients treated with CABG performed by the same surgeons between January 1, 2016 and December 30, 2019.

### Ethical statement

The study was approved by the institutional review boards at the Philipps University of Marburg, including a waiver of informed consent (ek_mr_110221_Wensauer-2).

### Study population

We reviewed 204 consecutive patients who underwent first time CABG for 3v-CAD with BIMA T-Graft as sequential grafting (C-T-BIMA) or with BIMA as T-Graft for the revascularization of the left-sided coronary arteries and an aorto-coronary graft for the right-sided vessels (L–T-BIMA + R-CABG, Fig. 1).

Exclusion criteria were: previous cardiac surgeries, concomitant carotid surgery, concomitant ablation for atrial fibrillation with or without left atrial auricular closure, higher degree (3rd grade) of mitral valve regurgitation (FMR) or tricuspid valve regurgitation (TR) preoperatively, concomitant valve or aortic surgery, and grafting with other BIMA configurations.

Four experienced consultant surgeons performed the procedures. Each surgeons applied both surgical techniques. The selection of the type of technique was at surgeon’s choice and was guided by patient characteristics. In emergency cases with life-threatening hemodynamical instability, in situ LIMA-LAD bypass was always performed; however, a shorter RIMA was harvested in order to reduce the duration of hemodynamic instability or/and CPB-time. As a result, these patients always required an additional graft, finally receiving L–T-BIMA + R-CABG revascularization. On the other hand, elective diabetic patients, who previously underwent venous stripping and were having inadequate diameter of the radial artery in ultrasonography, always received C-T-BIMA revascularization.

### Operative characteristics

All operations were performed under general anesthesia, via full sternotomy, using normothermic CPB and achieving cardiac arrest using either warm blood or cold crystalloid cardioplegia. All IMA grafts were harvested in skeletonized cold fashion, using clipping and minimal use of low-energy electrocautery. The RIMA was divided at its origin and connected end to side to the LITA as a T-graft. The in situ LIMA was always connected to the LAD. The radial arteries and the saphenous veins were harvested using an open surgical technique.

### Data collection

Relevant history and preoperative and postoperative study variables were obtained from the clinical records.

The MediStim Butterfly Model BF 2004 transit time flow meter (MediStim AS, Oslo, Norway) was used for direct intra-operative assessment of every graft after weaning from CPB.

Intraoperative bypass flow assessment was performed in a similar clinical setting in all patients: after weaning from CPB, under DDD-pacing at 80 beats/min, mean blood pressure of 50–55 mmHg, and hemoglobin above 8 dg/ml.

Echocardiographic assessment was obtained according to the current guidelines [18]. Cardiac function was determined by transthoracic echocardiography at hospital arrival and at hospital discharge. Transesophageal echocardiography was performed intraoperatively in all patients, monitoring cardiac unloading during CPB and determining myocardial and valve function before CPB and after weaning from CPB.

After hospital discharge, patients were receiving cardiological surveillance with transthoracic echocardiography yearly.

Pharmaceutical treatment including ß-blockers, ACE inhibitors, diuretics, and statins was applied in all patients in conformity with the current recommendations of the European Society of Cardiology on ischemic cardiac disease, arterial hypertension and functional mitral regurgitation. Medication was adjusted in all patients during follow-up based on the clinical, laboratory, and echocardiographic findings.

### Risk scores and definitions

Euroscore II and STS-score, Syntax score I (anatomical complexity), and Syntax score II for CABG (anatomical complexity, demographic, and clinical factors) were calculated as described elsewhere [19, 20]. Patients were subdivided by SS-value: group I (low or intermediate SS of ≤ 32), group II (high SS of > 32) for further analysis.

Revascularization was declared complete when the number of stenotic vessels (main coronaries or branch arteries) equaled the number of anastomoses [21].

The progression of FMR is defined as a relevant increase of the regurgitation volume caused by annular dilatation (Carpentier Type I) and/or left ventricular dilatation (Carpentier Type IIIb) without signs of degenerative disease and without new-onset of valve leaflet pathology.

Major adverse cardiac and cerebrovascular events (MACCE) were defined as death from any cause, nonfatal myocardial infarction, nonfatal stroke, and need for repeat revascularization.

### Outcome measures

Primary outcome measure is a comparison of the surgical results (bypass flow measurements and completeness of revascularization) and of clinical outcome at hospital discharge (complications, MACCE and all-cause mortality) of the two techniques.

Secondary outcome measure is the determination of the postoperative evolution of FMR and TR at follow-up.

Tertiary outcome measure consisted of identification of predictors for postoperative MACCE and FMR progression.

### Statistical analysis

Results are expressed as mean ± SD (standard deviation) for continuous variables and as percentages for categorical variables. Student's t-test, Kruskal–Wallis and Chi-Test evaluated differences between groups. Forward stepwise logistic regression and Cox regression were used to identify predictors for MACCE and FMR progression, respectively. Freedom from FMR progression was analyzed by Kaplan–Meier and compared by log rank test. A *p* value < 0.05 was considered significant. SPSS version 20.0 (IBM Corp Armonk, NY, USA) was used.

## Results

### Preoperative data

Baseline characteristics are listed in Table 1. Coronary angiography revealed more D_1_-Stenosis in C-T-BIMA patients (14% vs.6.7%, *p* = 0.044) and more RCA-Stenosis in L–T-BIMA + R-CABG patients (98% vs.84%, *p* = 0.003). Accordingly, less RCA-PCI was found in the L–T-BIMA + R-CABG group (4% vs. 14%, *p* = 0.013). Patients receiving L–T-BIMA + R-CABG suffered more from concomitant FMR and TR compared to C-T-BIMA (29.8% vs. 12%, *p* = 0.01, 14% vs.4%, *p* = 0.028, respectively). Risk scores were comparable between the groups.

**Table 1:** Preoperative data

	C-T-BIMA	L–T-BIMA + R-CABG	*P*-value
Patient Nr	100	104	
Pat. characteristics			
Age (years)	67.89 ± 10.04	66.08 ± 9.72	0.203
BMI (kg/m^2^)	28.35 ± 4.51	28.66 ± 4.96	0.608
Obesity (%)	33 (33)	45 (43)	0.351
Male gender (%)	83 (83)	81 (78)	0.833
Art. hypertension (nr/%)	89 (89)	90 (87)	0.210
Diabetes mellitus (nr/%)	42 (42)	38 (37)	0.347
COPD (nr/%)	6 (6)	16 (15)	0.059
Smoker (nr/%)	10 (10)	19 (18)	0.166
Alcohol abuse (nr/%)	1 (1)	1 (1)	1.000
Renal insufficiency (nr/%)	25 (25)	30 (29)	0.650
Clinical symptoms			
Angina pectoris (nr/%)	71 (71)	87 (84)	0.031
Dyspnoe (nr/%)	51 (51)	34 (33)	0.002
STEMI/NSTEMI (nr/%)	19 (19)	35 (34)	0.004
CPR (nr/%)	2 (2)	2 (2)	1.000
Decompensation (nr/%)	4 (4)	5 (5)	1.000
IABP/ECMO (nr/%)	0 (0)	3 (3)	0.247
Pre-OP lab. data			
Trop-I (mean/SD)	54.60 ± 203.91	73.03 ± 337.55	0.091
CK (mean/SD)	163.02 ± 182.85	176.35 ± 581.28	0.140
CK-MB (mean/SD)	9.82 ± 39.5	5.86 ± 39.79	0.080
LDH (mean/SD)	169.88 ± 68.99	196.39 ± 68.77	0.001
Creatinine (mean/SD)	1.12 ± 0.6878	1.11 ± 0.95	0.018
Echocardiography			
LV-EF% (mean/SD)	55.77 ± 10.68	55.49 ± 5.24	0.635
MVR I–II (nr/%)	12 (12)	31 (30)	0.002
TVR I–II (nr/%)	4 (4)	14 (14)	0.028
Coronary angiography			
Left main (nr/%)	30 (30)	43 (41)	0.125
LAD occlusion (nr/%)	13 (13)	9 (9)	0.229
RCA occlusion (nr/%)	24 (24)	31 (30)	0.351
LAD (nr/%)	93 (93)	92 (89)	0.092
RD1 (nr/%)	23 (23)	39 (38)	0.037
RD2 (nr/%)	4 (4)	3 (3)	0.663
RIM (nr/%)	14 (14)	7 (7)	0.088
RCX (nr/%)	78 (78)	84 (81)	0.890
RMS1 (nr/%)	30 (30)	23 (22)	0.151
RMS2 (nr/%)	6 (6)	5 (5)	0.707
RCA (nr/%)	84 (84)	102 (98)	0.003
RIVP (nr/%)	13 (13)	9 (9)	0.229
RPLD (nr/%)	14 (14)	8 (8)	0.148
Previous PTCA			
LAD (nr/%)	16 (16)	8 (8)	0.083
RCX (nr/%)	5 (5)	4 (4)	0.745
RCA (nr/%)	14 (14)	4 (4)	0.013
Risk scores			
Syntax score I	27.77 ± 7.87	27.18 ± 6.65	0.735
Syntax score II CABG	30.93 ± 10.95	30.79 ± 11.23	0.726
Euroscore II	2.32 ± 2.67	2.30 ± 2.06	0.384
STS score mortality	1.31 ± 2.44	1.16 ± 1.55	0.923
STS morbidity/mortality	7.75 ± 9.18	7.08 ± 5.25	0.696

^*^p < 0.05; **p < 0.01 delirium compared to non-delirium in the same group

^**^p < 0.01 Delirium compared to non-delirium in the same group

### Operative data and technical outcome

L-BIMA + R-CABG was more often performed urgently and required longer operation times (Table 2), resulting in longer intubation time and longer ICU stay (Table 3). More distal anastomoses, especially on the RCA system, were performed in the L-T-BIMA + R-CABG group leading to improved completeness of revascularization (84% vs. 69%, *p* = 0.014). Higher total bypass flows and increased flow/anastomosis were also achieved with L-T-BIMA + R-CABG (Table 2).

**Table 2:** Intraoperative data

	C-T-BIMA	L–T-BIMA + R-CABG	*P*-value
Patient Nr	**100**	104	
Type of operation			
Urgent (nr/%)	11 (11)	25 (24)	0.010
Elective (nr/%)	89 (89)	78 (76)	0.010
Type of cardioplegia			
Cold crystalloid (nr/%)	42 (42)	49 (47)	0.705
Warm blood (nr/%)	58 (58)	55 (53)	0.725
Operative times			
Operation (min)	248.3 ± 60.2	284.7 ± 66.0	0.0002
CPB (min)	92.7 ± 21.8	117.0 ± 34.2	0.0000
Cardioplegic arrest (min)	70.2 ± 18.4	82.5 ± 21.1	0.0000
Type of coronary grafting			
BIMA	100 (100)	–	–
BIMA + RIMA segment	–	21 (20)	–
BIMA + A. radialis	–	21 (20)	–
BIMA + GSV	–	62 (60)	–
Revascularization			
Completeness of revasc	69 (69)	87 (84)	0.014
Nr. of distal anastomoses	3.71 ± 0.69	4.02 ± 0.87	0.015
To LAD system	1.46 ± 0.52	1.41 ± 0.53	0.464
To RCX system	1.23 ± 0.49	1.39 ± 0.57	0.059
To RCA system	1.02 ± 0.32	1.21 ± 0.43	0.001
Flow measurements			
Total flow of all grafts	82.50 ± 49.26	125.88 ± 92.41	0.000
Flow per anastomosis	22.77 ± 14.23	31.83 ± 23.90	0.001
Flow of the T-graft	82.50 ± 49.26	61.88 ± 51.30	0.005
Flow of RCA grafts	–	63.97 ± 63.34	
Total flow on LAD system	21.86 ± 12.46	32.83 ± 36.03	0.000
Total flow on RCX system	8.8 ± 0.78	20.84 ± 32.23	0.000
Total flow on RCA system	4.50 ± 0.55	63.84 ± 63.47	0.000

**Table 3:** Postoperative data

	C-T-BIMA	L-T-BIMA + R-CABG	*P*-value
Patient Nr	100	104	
Post-OP times			
Intubation time (h)	16.28 ± 25.63	20.60 ± 18.29	0.000
ICU time (d)	2.94 ± 2.91	3.10 ± 3.20	0.000
Hospital stay (d)	13.03 ± 5.25	12.40 ± 8.53	0.002
Post-OP lab. Data			
CK max	1134.27 ± 1124.21	1064.62 ± 1010.12	0.672
CK-MB max	44.79 ± 43.05	50.92 ± 40.92	0.029
Creatinine max ()	1.31 ± 0.91	1.37 ± 1.08	0.845
Post-OP complications			
ECMO/IABP on ICU	0 (0)	1 (1)	1.000
CPR on ICU	2 (2)	0 (0)	0.239
Re-do for bleeding	0 (0)	2 (1.9)	0.164
Re-do for bypass-revision	2 (2)	1 (1)	0.539
Sternal infection	15 (15)	15 (14.4)	0.438
Saphenectomy infection	–	2 (1.9)	–
Stroke	3 (3)	5 (4.8)	0.498
Delirium	20 (20)	17 (16.3)	0.499
Tracheotomy	1 (1)	1 (1)	0.978
Post-OP PCA/PTCA (FU)			
Coronary angiography	2 (2)	4 (3.8)	0.683
PCI ± stenting	0 (0)	4 (3.8)	0.122
Echocardiography (FU)			
LV-EF% (mean/SD)	57.76 ± 7.71	57.22 ± 6.23	0.128
Post-OP MVR °I–II (nr/%)	64 (64)	49 (47)	0.017
Post-OP TVR °I–II (nr/%)	22 (22)	12 (12)	0.059
Outcome (mortality)			
MACCE at 30 days	8 (8)	2 (1.9)	0.055
Mortality at 30 days	2 (2)	1 (0.96)	0.617
Mortality at FU	4 (4)	6 (5.8)	0.748

### Clinical outcome

Early complications, MACCE, mortality at 30 days and at follow-up were similar between techniques (Table 3). LV function at follow-up improved in the L-BIMA + R-CABG group (57.22 ± 6.23 vs. 55.49 ± 5.48 preoperatively, *p* = 0.035). There was a maximum postoperative follow-up time of 5 years and minimum follow-up time of 24 month.

Although more patients in the L-T-BIMA + R-CABG group suffered from FMR preoperatively (30% vs. 12% *p* = 0.002), postoperative FMR progression was lower in the L-T-BIMA + R-CABG group (47% vs. 30%, *p* = 0.0152) than in C-T-BIMA (64% vs.12%, *p* < 0.0001).

**Fig. 2:**
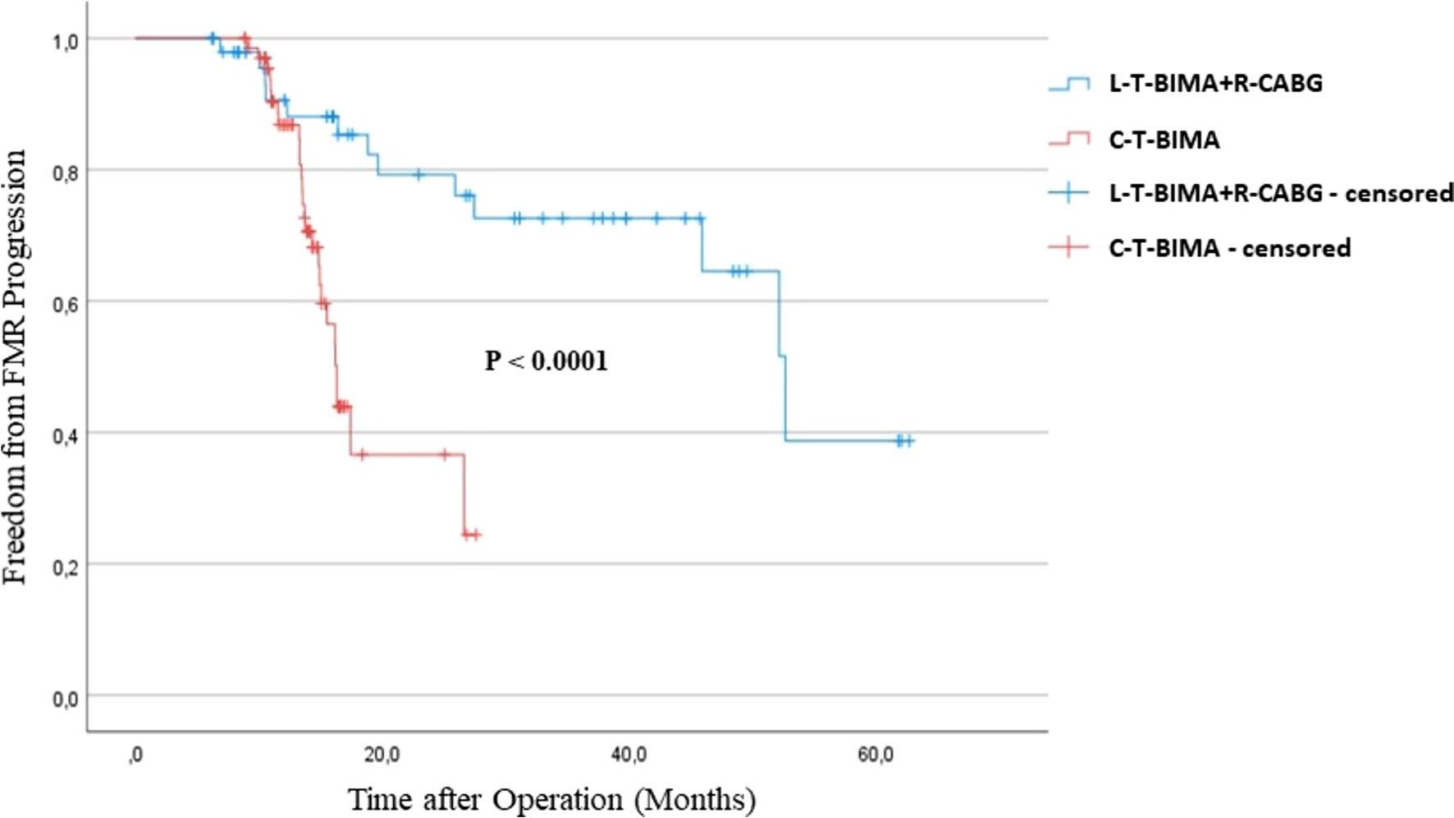
Freedom from progression of FMR after CABG for 3v-CAD. Kaplan–Meier curve of freedom from FMR progression after sequential composite grafting for 3v-CAD with L-T-BIMA + R-CABG and C-T-BIMA

**Fig. 3:**
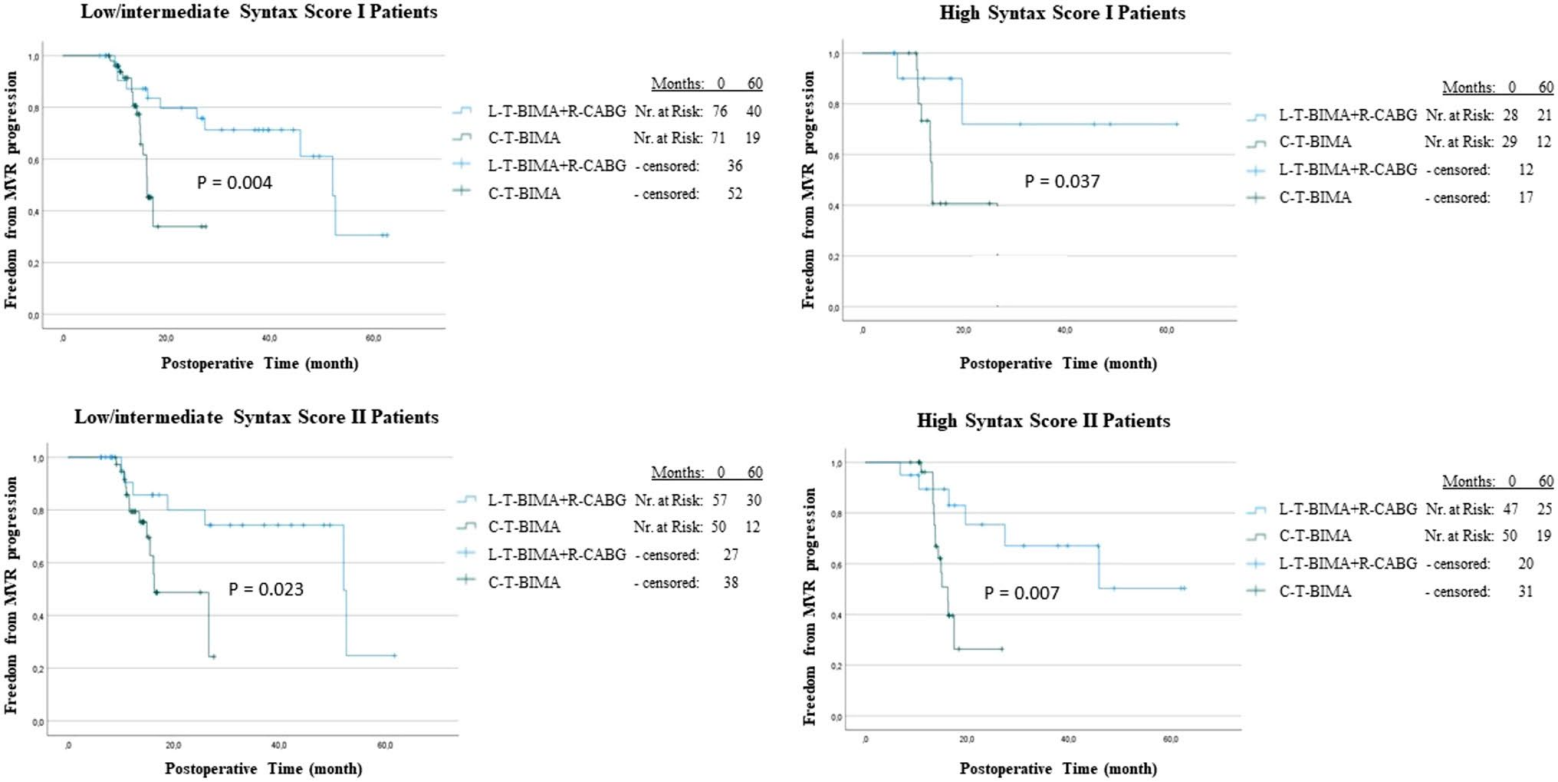
Freedom from progression of FMR after CABG redeployed by Syntax Scores. Kaplan–Meier curve for freedom from FMR progression stratified by the type of surgery in patients with low/intermediate SS I, high SS I, low/intermediate SS II and high SS II.

The Kaplan–Meier analysis revealed pronounced FMR progression in the C-T-BIMA group vs. L–T-BIMA + R-CABG (Fig. 2), independent of Syntax Scores (Fig. 3).

TR was higher in the L-T-BIMA + R-CABG than in C-T-BIMA (14% vs. 4%, *p* = 0.028) before the operation (Table 1), progressed postoperatively in the C-T-BIMA group (22% vs. 4%, *p* = 0.0002) and tended to decrease in the L-BIMA + R-CABG group (12% vs. 14%, *p* = 0.8943).

### Risk factor analysis

No risk factors for MACCE were identified in our study (Supplemental Table 1).

Preoperative RCA occlusion (HR = 3.0, *p* = 0.023) and the type of operation (HR = 4.27, *p* = 0.010) influenced FMR progression in the entire cohort (Table 4), with RCA occlusion (HR = 9.932, *p* = 0.006), preoperative LV-EF (HR = 0.858, *p* = 0.019) and patient age (HR = 1.257, *p* = 0.012) predicted FMR progression in C-T-BIMA patient subgroup (Table 5, 6). *P*-value < 0.05 is statistically singificant.

**Table 4:** Logistic regression analysis of all variables as predictors for MACCE

Variables	OR	SD	95% CI		VIF	*P*-Value
			LL	UL		
Pre-OP scores						
Syntax score I	−0.006	0.007	−0.020	0.009	1.484	0.443
Syntax score II	0.007	0.006	−0.005	0.020	3.082	0.233
Euroscore II	−0.037	0.046	−0.127	0.054	3.887	0.421
STS mortality score	−0.012	0.056	−0.123	0.100	4.222	0.834
STS mort. and morbid. Score	0.014	0.024	−0.034	0.062	8.070	0.563
Pre-OP variables						
Age	−0.006	0.007	−0.020	0.009	3.013	0.440
Pre-OP LM disease	−0.125	0.114	−0.352	0.102	1.803	0.276
Pre-OP LAD occlusion	−0.042	0.153	−0.347	0.263	1.338	0.786
Pre-OP RCX occlusion	0.154	0.095	−0.034	0.342	1.266	0.107
Pre-OP RCA occlusion	−0.077	0.112	−0.301	0.147	1.389	0.495
Pre-OP LV-EF (%)	0.003	0.005	−0.008	0.013	1.928	0.620
Pre-OP MVI > I°	−0.038	0.108	−0.252	0.176	1.349	0.726
Pre-OP creatinine	0.064	0.187	−0.307	0.436	1.840	0.732
Pre-OP LDH	0.002	0.001	0.000	0.004	2.407	0.092
OP characteristics						
CPB time	0.000	0.004	−0.009	0.008	8.000	0.909
Cardiac arrest time	0.000	0.006	−0.011	0.012	8.022	0.956
Type of cardioplegia	−0.029	0.092	−0.211	0.154	1.232	0.754
Nr anast LAD system	0.104	0.165	−0.224	0.433	4.383	0.530
Nr anast RCX system	0.001	0.149	−0.295	0.298	3.344	0.993
Nr anast RCA system	0.251	0.186	−0.119	0.620	2.916	0.181
Operation type	0.015	0.116	−0.216	0.246	2.039	0.898
Completeness of revasc	−0.058	0.110	−0.277	0.161	1.371	0.602
Total flow of all grafts	−0.001	0.004	−0.009	0.007	57.289	0.829
Bypass flow/anast	0.005	0.016	−0.027	0.038	57.546	0.749

**Table 5:** Part I COX regression analysis of predictors for MI progression in the entire cohort

Variables	B	HR	95% CI		Wald	*P*-Value
			LL	UL		
Pre-OP scores						
Syntax score I	0.005	1.005	0.944	1.070	0.026	0.871
Syntax score II	−0.013	0.987	0.926	1.052	0.164	0.685
Euroscore II	0.127	1.135	0.627	2.054	0.303	0.675
STS mortality score	−0.377	0.686	0.158	2.975	0.748	0.615
STS mort. and morbid. Score	−0.039	0.962	0.711	1.301	0.154	0.799
Pre-OP variables						
Age	−0.690	0.502	0.115	2.188	0.843	0.359
Pre-OP LM disease	0.450	1.568	0.539	4.563	0.681	0.409
Pre-OP LAD occlusion	0.669	1.952	0.649	5.873	1.417	0.234
Pre-OP RCX occlusion	−0.128	0.880	0.376	2.057	0.087	0.768
Pre-OP RCA occlusion	1.101	3.006	1.166	7.750	5.188	0.023
Pre-OP LV-EF (%)	−0.020	0.980	0.896	1.072	0.194	0.660
Pre-OP LV-EF < 40%	0.327	1.387	0.110	17.519	0.064	0.800
Pre-OP MVI > I°	−0.353	0.703	0.120	4.113	0.153	0.695
OP characteristics						
CPB time	0.006	1.006	0.968	1.045	0.082	0.774
Cardiac arrest time	0.010	1.010	0.962	1.061	0.173	0.678
Type of cardioplegia	0.982	2.669	0.672	10.607	1.945	0.163
Nr Anast LAD SYSTEM	−0.653	0.520	0.133	2.042	0.877	0.349
Nr Anast RCX system	−0.003	0.997	0.232	4.289	0.000	0.997
Nr Anast RCA system	0.290	1.337	0.191	9.365	0.086	0.770
Operation type	1.451	4.268	1.407	12.944	6.571	0.010
Completeness of revasc	−0.327	0.721	0.250	2.079	0.367	0.545
Bypass flow/anast	−0.026	0.974	0.812	1.169	0.079	0.779

**Table 6:** Part II COX regression analysis of predictors for MI progression in the subgroups

Variables	B	HR	95% CI		Wald	*P*-Value
			LL	UL		
C-T-BIMA						
Age	0.229	1.257	1.052	1.502	6.312	0.012
Pre-OP RCA occlusion	2.296	9.932	1.926	51.206	7.527	0.006
Pre-OP LV-EF (%)	−0.153	0.858	0.755	0.975	5.501	0.019
Syntax score II	−0.109	0.897	0.802	1.003	3.632	0.057
L–T-BIMA + R-CABG						
Age	0.835	2.304	0.444	11.946	0.988	0.320
Pre-OP RCA occlusion	7.338	1536.91	0.003	70.361	1.217	0.270
Pre-OP LV-EF (%)	1.768	5.860	0.567	60.585	2.201	0.138
Syntax score II	−0.842	0.431	0.082	2.259	0.992	0.319

## Discussion

This study reveals that the adoption of the two-inflow L-T-BIMA + R-CABG technique in patients undergoing CABG for 3v-CAD is associated with improved completeness of revascularization when compared to the one-inflow C-T-BIMA grafting, without increasing the incidence of MACCE and mortality.

In addition, the L-T-BIMA + R-CABG impeded the progression of both FMR and TR more effectively than the C-T-BIMA revascularization technique.

Beyond expectations, in this cohort of largely uncomplicated CABG patients, total bypass flow increased with the number of the distal anastomoses, with the completeness of revascularization and with the number of inflows (Table 2). The Cox analysis (Table 4) disclosed a relative importance of bypass flow measurement, showing that neither the amount of bypass flow nor completeness of revascularization or the number of distal anastomoses were able to predict the progression of FMR after CABG. This discrepancy can be explained by the fact that the flow per each distal anastomosis was not measured but calculated in our study. Sequential bypass makes measurement between the distal coronary anastomosis difficult and does not allow direct quantification of the flow per anastomosis. Bypass flows change over time depending on the graft size, inflow, and peripheral resistance of the myocardial territory, with the later also changing after surgery, in direct proportion to the amount of the initially under-perfused myocardium that is recruited after revascularization.

Thus, the type of surgical technique and the presence of RCA occlusion proved to be the only predictors of FMR progression in 3v-CAD patients undergoing CABG (Tables 5, 6). In particular, patients with RCA occlusion benefit from the two-inflow revascularization. Presumably, the two-inflow L-T-BIMA + R-CABG technique, that is associated with a significantly higher number of distal anastomoses on both RCX and RCA coronary systems (Table 2), generates a significant augmentation of blood flow not only to the postero-inferior, but also to the left-lateral wall, thus improving the perfusion of both papillary muscles of the mitral valve. The multiple distal anastomoses on the terminal branches of the RCA system (PDA and RPL) additionally enhance the perfusion of the interventricular septum and posterior wall and thus improve left-side heart function, finally resulting in a relevant reduction of the tricuspid valve regurgitation.

These findings are in agreement with previous studies [6, 8, 9] revealing that competition of flow plays a crucial role in the long-term arterial grafting functionality, especially when sequential grafting is applied, and that the competitive flow occurring in sequential grafting is minimized when two inflows are applied. Thus, our results highlight that beyond completeness of revascularization, graft configuration is the most important factor influencing myocardial perfusion after CABG.

Thus, the hereby evaluated L-T-BIMA + R-CABG technique condenses the advantages of the fully arterial revascularization for the left-sided coronaries [6, 9] and offers a separate inflow for the revascularization of the right-sided coronary branches [14, 22, 23], resulting in outright myocardial perfusion.

First, these findings are in agreement with previous meta-analyses [24–26] demonstrating that BIMA grafting is associated with a significant reduction in early mortality, with improved long-term survival and with reduced risk of repeat revascularization compared to LIMA, without any adverse effects. In situ BIMA has been previously proved to provide greater bypass flow that in situ LIMA, especially when more than five branches were targeted [6, 9, 10], with the amount of bypass flow increasing proportionally with the number of distal anastomoses [5]. Moreover, there are both randomized [27, 28] and observational evidence [22, 23] demonstrating that composite grafting does not compromise graft patency or survival, probably because IMAs are the best-equipped arterial conduit to withstand the competition flow because of their endothelial function [25]. In situ LIMA also has the capacity to dilate to meet the local demands and even improves over time [26]. Furthermore, using the T-graft is not associated with a potential loss in the LIMA to LAD component of the graft [12, 13] and a patent LIMA to LAD affords significant benefit in terms of patient survival and freedom from MACCE [24–26, 29]. On other note, the use of BIMA as T-graft is associated with a reduced need for repeated revascularization [23, 26] and has been shown to provide an additional survival benefit and freedom from MACCE in patients undergoing CABG [14, 15].

Second, the use of a separate aortic inflow in the L-T-BIMA + R-CABG group is not a prognostic factor for MACCE. In agreement with previous studies [25, 28, 30], our data reveal that neither risk scores or type of operation, nor the number of distal anastomoses correlated with MACCE in CABG patients.

These findings may be linked to a well-conceived diagnostic and surgical strategy. Shortly, all our patients were scanned preoperatively for carotid artery stenosis. If stenosis was present, it was treated at the time of the CABG operation and these patients were excluded from the study. All patients suffering from atrial fibrillation received LAA closure with or without ablation at the time of CABG surgery, and atrial fibrillation was an exclusion criterion of our study. In addition, all patients over 70 years old and those with left main coronary artery disease received a thoracic CT scan for the preoperative evaluation of the aortic calcification. Intraoperative aortic cannulation was performed in Seldinger technique under TEE guidance. This overarching strategy could explain why the completion of an aortic inflow in the L-T-BIMA + R-CABG group did not increase the incidence of MACCE. Another reason is the preoperatively low risk profile of our cohort (Table 1). Scores that include clinical and angiographic variables, such as SS II, and global risk score such as Euroscore II and STS scores proved to be more suitable as predictors for MACCE than the purely angiographic SS I [23, 24]. In our study, there were no significant differences between the preoperative SS I and SS II of the two patient subgroups. The STS scores and Euroscore II were also similarly low (Table 1). Alike other studies [19, 20, 30], we found that risk scores, the type of operation, and the number of distal anastomoses did not correlate with MACCE in CABG patients (supplemental Table 1).

Notably, patients receiving L-T-BIMA + R-CABG showed a reduction in all-cause FMR progression at 5-year follow-up when compared to C-T-BIMA technique that was achieved with no increase in postoperative mortality or morbidity.

Whereas type of surgery and the preoperative RCA occlusion predicted FMR progression after CABG in the entire cohort (Table 4), LV-EF and RCA occlusion increased the risk of FMR progression only in the C-T-BIMA patients.

Comparison of freedom from progression of FMR in the two groups according to SS categories showed lower protection with C-T-BIMA technique in both—low–intermediate and high—SS I as well as SS II categories (Fig. 2). Thus, the L-T-BIMA + R-CABG technique performs better than C-T-BIMA in all aspects of postoperative outcome and independent of the SS scores.

In agreement with previous studies [19, 20, 30], our results also emphasize that SS scores alone cannot be utilized to guide the choice of surgical revascularization in patients with 3v-CAD.

## Limitations

Although the techniques were equally used at both institutions by the same surgeons, techniques selection bias by surgeons may be present.

Differences in ventricular diameters and volumes, segmental dysfunctions, tenting heights and areas might have strengthened the study; however, they could not be extracted for all patients.

Statistical interrogation of a larger database might next confirm the present findings.

## Conclusion

The L-T-BIMA + R-CABG revascularization technique that provides two separate inflows for the left- and right-sided grafting preserves all benefits of the BIMA T-graft revascularization without increasing the risk for MACCE, improves completeness of revascularization, and reduces the mid-term progression of FMR independent of the preoperative clinical and anatomical risks.

Older patients with reduced LV-EF, RCA occlusion, and higher Syntax-scores benefit most from the two-inflow L-T-BIMA + R-CABG technique.

Younger patients with 3v-CAD and normal LV-EF can preferentially be managed with the one-inflow C-T-BIMA; however, the long-term outcome remains to be revealed.

## Data Availability

The raw data that support the findings of this study are available from the corresponding author [T.B.A.], upon reasonable request.

## Abbreviations

3v-CAD: Three-vessel coronary artery disease
CABG: Coronary artery bypass grafting
LIMA: Left internal mammary artery
BIMA: Bilateral internal mammary artery
LAD: Left anterior descending coronary artery
C-T-BIMA: Complete revascularization with both internal mammary arteries as T-graft
L-T-BIMA: Left-sided revascularization with both internal mammary arteries as T-graft
R-CABG: Right-sided coronary artery bypass grafting with an aorto-coronary graft
RCA: Right coronary artery
LV-EF: Left ventricular ejection fraction
CT-scan: Computer tomographic scan
TEE: Transesophageal echocardiography
MACCE: Major adverse cardiac and cerebrovascular events
FMR: Mitral valve regurgitation
TR: Tricuspid valve regurgitation
SS: Syntax (synergy between PCI with taxus and cardiac surgery) Score
Syntax: Synergy between PCI with taxus and cardiac surgery
MRI: Magnetic resonance imaging
